# Sexual dimorphism through androgen signaling; from external genitalia to muscles

**DOI:** 10.3389/fendo.2022.940229

**Published:** 2022-07-27

**Authors:** Lerrie Ann Ipulan-Colet

**Affiliations:** College of Science, Institute of Biology, University of the Philippines, Diliman, Quezon City, Philippines

**Keywords:** androgen, muscle, external genitalia, sexual dimorphism, developmental mechanism

## Abstract

Sexual dimorphisms can be seen in many organisms with some exhibiting subtle differences while some can be very evident. The difference between male and female can be seen on the morphological level such as discrepancies in body mass, presence of body hair in distinct places, or through the presence of specific reproductive structures. It is known that the development of the reproductive structures is governed by hormone signaling, most commonly explained through the actions of androgen signaling. The developmental program of the male and female external genitalia involves a common anlage, the genital tubercle or GT, that later on develop into a penis and clitoris, respectively. Androgen signaling involvement can be seen in the different tissues in the GT that express Androgen receptor and the different genes that are regulated by androgen in the mesenchyme and endoderm component of the GT. Muscles are also known to be responsive to androgen signaling with male and female muscles exhibiting different capabilities. However, the occurrence of sexual dimorphism in muscle development is unclear. In this minireview, a summary on the role of androgen in the sexually dimorphic development of the genital tubercle was provided. This was used as a framework on analyzing the different mechanism employed by androgen signaling to regulate the sexual dimorphism in muscle development.

## Introduction

Biological differences are evident in extant species to date, accounting for great diversity in physiology, reproductive strategies, anatomy, and morphology. Initially, dimorphic organs have similar precursor structures that undergo the same early developmental program which includes mitotic growth and cellular differentiation. This will then be followed by a stage of divergent developmental process which usually involves sex hormones, androgens and estrogens, which leads to sexual dimorphism. This sexual dimorphism is important in establishing male- and female-specific organs to ensure greater reproductive success. Among the apparent manifestations of sexual dimorphism can be determined morphologically in terms of size, shape, colors, and development of appendages (1). In many mammalian species such as elephants, sea lions, and kangaroos, adult males are larger than females. Neck crests are also prominent in male bulls and stallions due to greater muscle build-up in the head, neck, and shoulder regions as compared to their female counterparts and has been attributed to androgen-facilitated muscle deposition patterns (2). However, the developmental program underlying sexual dimorphisms are not yet fully understood.

One of the most notable sexually dimorphic structures in mice are the features of the external genitalia (ExG) and the anogenital distance (AGD) in the perineum, area between the anus and genitals (3). Male mice have longer and more pronounced ExG and AGD as compared to female mice. Some distinguishing features of the ExG between the males and females include the presence of erectile tissue (diffused in clitoris), organ size (larger for the penis), bone (larger for the penis), cartilage (absent in the clitoris), surface spines (absent in the clitoris), urethral position (completely within the penis for males), and cross-sectional shape (U-shaped for the clitoris and circular for the penis) (4). The penis and clitoris of the ExG both develop from the ambisexual genital tubercle (GT), from embryonic day 11 or E 11, and become sex-specific at E 16 in mice (5). The longer AGD is used in sexing mice pups as early as E16.5. The perineum region is longer in male mice which may be attributed to the presence of well-developed bulbocavernosus/levator ani (BC/LA) muscle complex (3).

Androgen signaling has been identified as the primary factor that establishes male characteristics. The ligands for androgen signaling are testosterone (T) and dihydrotestosterone (DHT). T is the primary circulating androgen that is secreted by the Leydig cells of the testis while DHT is a product of the conversion of T *via* 5a-reductase in several reproductive tissues (6). The androgen receptor (*Ar*) is composed of different functional domains: N-terminal domain, DNA-binding domain, hinge region and C-terminal or ligand binding domain (7). Androgen signaling is initiated by the binding of androgens, (T) or dihydrotestosterone (DHT) to androgen receptor resulting to the dimerization of Ar and its nuclear translocation. The Ar can bind to the DNA of target genes through androgen response elements (ARE) (8). Ar is known to regulate transcription of different genes and more recently, non-genomic functions of AR have been evident (ADD 2 here).

The developmental program of the ExG formation has been explained through the dominant effect of androgen signaling. Muscle development and physiology is known to be responsive to the effect of testosterone. The understanding of the masculinization process of the external genitalia and AGD might help in identifying the occurrence of sexually dimorphic muscle development.

## The study of sexual dimorphism in the external genitalia

**Figure 1:**
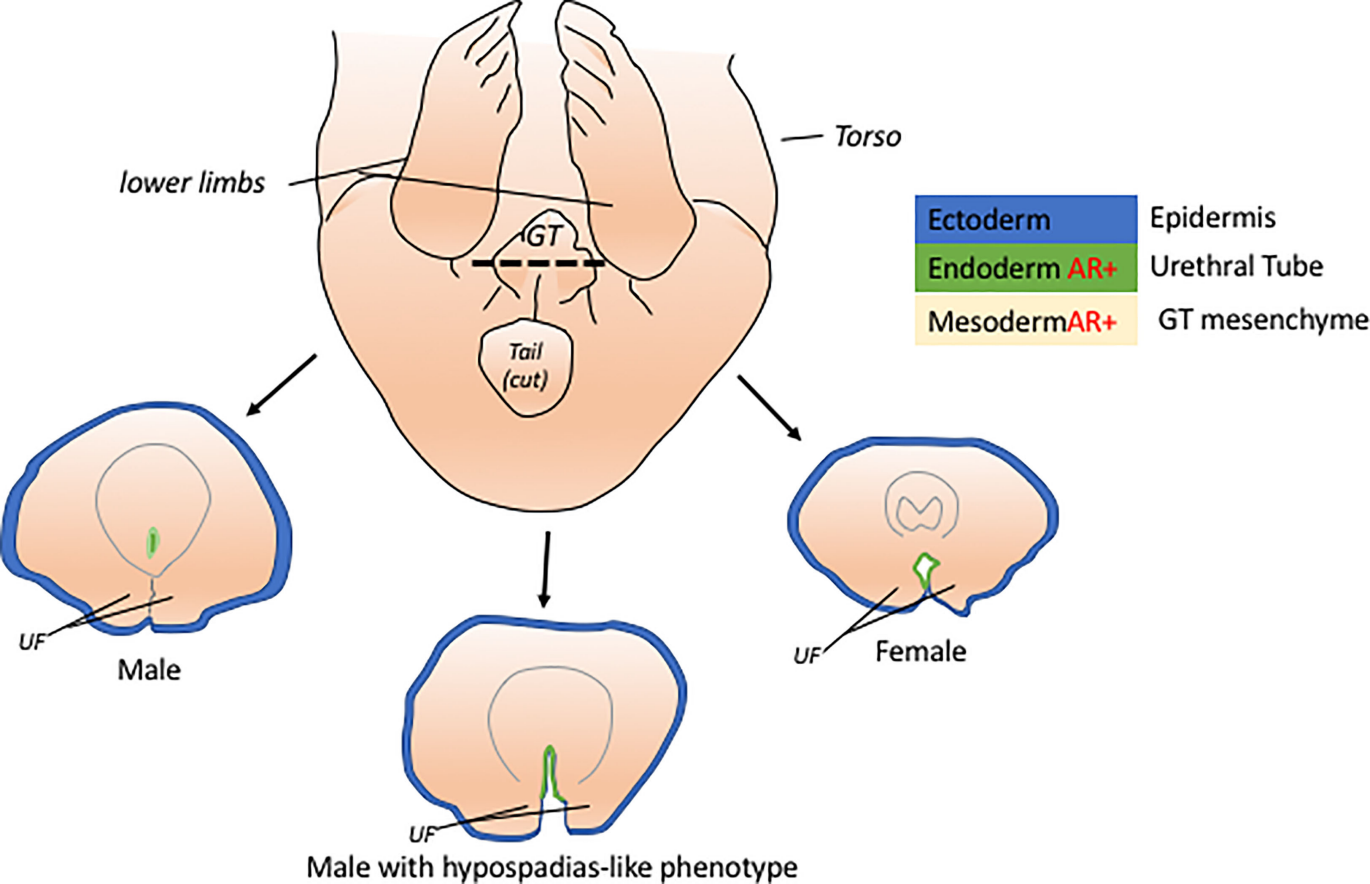
The genital tubercle or GT is the common anlage of the penis and clitoris. The GT has cells from 3 embryonic layers, ectoderm, mesoderm, and endoderm. At early embryonic stage, the male GT is distinguished from female GT through the fusion of the urethral folds (UF) which can be viewed histologically through a cross-section of the GT (dotted line). The masculinization of the male GT is attributed to the active androgen signaling in the endoderm and mesoderm tissues of the GT. Unfused UF in males, hypospadias-like phenotype, are due to disruptions in the signaling of the endoderm and mesoderm of the GT.

The development of the ExG starts with a common anlage known as the genital tubercle or GT (**Figure 1**) which will later develop into the penis and clitoris. The GT is composed of different tissues originating from the ectoderm, endoderm, and mesoderm. The epidermis of the GT is mainly comprised of ectodermal cells while the urethral tube is of endodermal origin. The mesenchyme of the GT is derived from the pericloacal mesenchyme which is primarily from the mesoderm. Each of these tissue types contribute to the development of the GT and towards a sexual dimorphic structure of a penis or clitoris. One of the most notable structure that distinguish the male and female GT is the fusion of urethral folds. Failure of urethral folds fusion leads to hypospadias, a human penile condition wherein the urethral opening is not located at the tip of the penis. This condition can be replicated in mice when androgen signaling is disrupted during the stage of urethral tube fusion (9, 10). Additionally, exclusive dysregulation of signaling in the ectoderm, endoderm,or mesenchyme exhibits a hypospadias-like phenotype, in varying degree, due to the failure in the proper fusion of the urethral folds (10–13). This shows that each of this tissue contributes to GT masculinization.

Androgen signaling has been a consistent variable in the study of a masculinized GT. Androgen receptor (AR) expression is observed in the mesenchyme of the urethral folds and the urethral tube while its expression in the ectoderm is not detected (10, 12). Only the mesenchyme -specific knock-out (KO) of AR results to hypoplasia of the urethral folds. This indicates that the influence of androgen signaling during male-specific GT development may depend on which AR-expressing tissue is most important in the masculinization process (ADD 10 or 12). A microarray analysis comparing the GT ventral mesenchyme and urethra of male and female shows some genes that are differentially expressed at the onset of sexually dimorphic features. *Cyp1b1*, *Fkbp51* (FK506 binding protein 5) and *Mafb* (v-maf masculoaponeurotic fibrosarcoma oncogene family protein B) are differentially expressed in male and female GTs (14). *Mafb* is more prominently expressed in the male GT mesenchyme than in females. Additionally, exogenous exposure to androgen induced *Mafb* expression in females while ArKO (Androgen receptor knockout) mice downregulated its expression. Interestingly, androgen exposure in the absence of *Mafb* resulted to failed masculinization of the urethra and *Mafb* KO mice exhibited defective urethra which is similar to hypospadias (10). This implies that androgen is essential in inducing and regulating Mafb action in the masculinization of the urethral folds. Another signaling pathway that is influenced by androgen signaling is the Wnt signaling. Beta-catenin (*B-cat*), a downstream effector of Wnt-signaling, is found to be highly expressed in the male ventral mesenchyme. Such expression pattern can be replicated in the female through the administration of testosterone propionate, a testosterone analogue. KO of *Ar* and *B-cat* in the urethral ventral mesenchyme both leads to hypospadias-like phenotype (12).

From the studies of GT development mentioned above, there are some investigative strategies that can be used as a framework in identifying sexually dimorphic muscle formation. First, it is important to identify which cell or tissues are necessary for the proper sexually dimorphic development of tissues or organs. And also, which of these tissues express *Ar* and which tissue-specific *Ar* deletion results to drastic changes in phenotype. Second, all the above-mentioned studies include comparison between male and female structures and differential gene expression patterns. This method is necessary to elucidate which developmental mechanism is necessary for general development of structures and which is necessary for male- or female- specific developmental program. It is important that identified mechanism may be used to manipulate the female GT development which will result to structures or expression pattern that will resemble that of the male, or vice versa. And lastly, genes that are differentially expressed between male and females need to be validated on how androgen signaling might influence its expression pattern. This can be done by using different hormonal modulation experiments (see Diff review paper) and analyze how gene expression patterns are affected.

## Muscle development

Muscle development starts with the specification of a somite region known as dermomyotome which leads to primary myotome formation. The myotome contains muscle cells or myoblasts that migrate to different parts of the body to serve as muscle precursor cells for the rib and back muscles, body walls, limbs and tongue. This process occurs between E9 – E12 in mice (15). During this time, the development of the muscles is not observed to be androgen-dependent nor is it different between male and female embryos.

**Figure 2:**
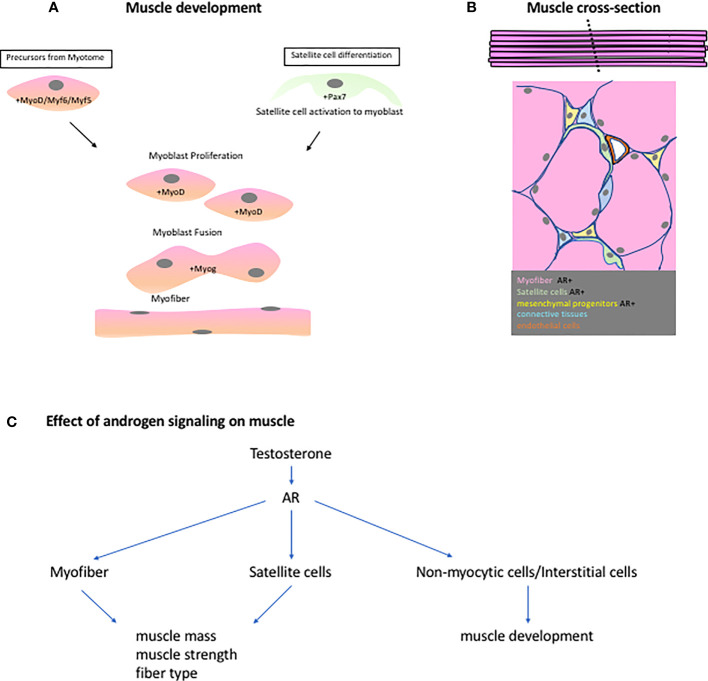
**(A)** Muscle embryonic development is highly regulated by the MRFs (Myf5, Myf6,MyoD and Myog). Precusor myoblast from the myotome travel to different parts of the body to establish the different muscle groups. These precursors or myoblast, expressing MyoD, will proliferate, fuse, and differentiate to form syncytial myofibers. During regeneration, the resident stem cells of the muscles, satellite cells, are activated and begin to express MyoD. It will then follow the same steps as in the embryonic muscle development. **(B)** A cross-section of the muscle shows the different cell population found in the muscles. Some of these cells express AR and are instrumental to the androgen-responsive nature of some muscle groups. **(C)** A scheme showing the effects of androgen signaling based on ArKO studies on different muscle groups.

The regulation of myogenesis is dependent on the expression of members of the basic helix-loop-helix domain-containing myogenic regulatory factors (MRFs) (**Figure 2A**). MRFs include myogenic factor 5 (*Myf5*) and 6 (*Myf6*), myogenic differentiation 1 (*MyoD*), and myogenin (*Myog*) (16). *Myf5* and *MyoD* are suggested to act redundantly and upstream of both *Myf6* and *Myog*. *Myf5* mutants exhibit initial delayed muscle development which was rescued once *MyoD* begins to be expressed resulting in an overall normal myogenesis (17, 18). Similarly, *MyoD* mutants possess normal skeletal muscle development with a notable persistent expression of *Myf5*, which is normally down-regulated after day 14 of gestation (18). This suggests that some functional redundancy between *MyoD* and *Myf5* exists. *Myog* null mutation results in normal initiation of myogenesis but defects in myocyte differentiation and myotube formation were observed (19, 20). These MRFs act downstream of the paired domain and homeobox-containing transcription factors paired box gene 3 (*Pax3*) and 7 (*Pax7*) (21, 22). *Pax3* mutants display failure in the proper specification of the dermamyotome and myotome compartments of the somite which may lead to defective body wall musculature development (23). *Pax 7* could substitute for some of the function of Pax3 in somite development but not the functions on myogenic specification (24). The primary role of *Pax7* is suggested to be implicated with the specification of myogenic satellite cells (22). The satellite cells contribute to the development, growth and repair of muscles. During development and muscle regeneration, the satellite cells are activated and start to express *MyoD* (**Figure 2A**). Activated satellite cells become the myoblasts and undergo proliferation. Post-mitotic myoblasts will further differentiate and express *Myog* or *Myf6*. These transcription factors will regulate the terminal differentiation of myocytes and myotube formation (25, 26). The regulatory mechanism of myogenesis is considered as one of the active areas of research and more findings are being accumulated that could specify the exact hierarchy and players during muscle development.

## Androgen effects on muscle development

The most recognized effect of androgens on muscles is studied through physiological changes on athletes taking testosterone (T) and other related steroids. It was discovered that intramuscular injection of T results in an increase in the net protein synthesis followed by enlargement of the muscle fibers (27). The mechanism of hypertrophy is explained through the concepts of nuclear domain and ceiling theory. A muscle fiber contains numerous myonuclei that sustain protein synthesis over a finite number of cytoplasm (nuclear domain). Upon T treatment, the protein synthesis becomes too high to be sustained by one myonuclei (ceiling theory) hence promoting myonuclear accretion. This process will create an enlargement of the muscle fiber leading to muscle hypertrophy (28–30). This could possibly be attributed to the ability of testosterone, through the androgen receptor, to increase the expression of *Myog* and *mTOR* and decrease the expression of myostatin, a negative regulator of muscle mass (31). Additionally, it has been reported that androgen can regulate the expression of polyamine biosynthesis genes such as *Odc1* and *Amd1*. This may also mediate the anabolic actions of androgens on muscle mass (32).

To understand the effect of androgen on the sexual dimorphism in muscle development, the points discussed in studying sexual dimorphism in GT can be used as perspective for summarizing the role of androgen on muscles. First is to identify the type of tissues that are necessary for muscle development and how Ar is expressed and utilized in those cells. A summary of this can be seen in **Supplementary Table 1**. The effect of androgen signaling is very diverse since the muscle cell population is also composed of different types of cells (**Figure 2B, C**). A cross section of a skeletal muscle will reveal the presence of different cell such as myofibers, satellite cells, connective tissues, pericytes, endothelial cells, and other mesenchymal cells.

Satellite cells (SC) are the resident stem cells of the muscles and testosterone treatment results in the increase of SC number in humans, rats and pigs leading to muscle hyperthrophy (33–36). SC are described as targets of androgen actions since *Ar* expression is evident on SC in the LA muscle (37). In a study using depletion of satellite cells and testosterone treatment, it was discovered that muscle hypertrophy occurred in testosterone-treated female mice regardless if the mice were depleted with SC (*Pax7^CreER/CreER^+;Rosa26^DTA/DTA^*) or with the normal number of SC (38). This was further confirmed when satellite cell-specific AR KO (*Pax7^CreERT2^+;AR^flox^*)female mice exhibited increased mass upon testosterone treatment (39). In the same study, a myofiber-specific ArKO or mArKO (*Ar^flox/flox^;HSA-Cre*) was observed to have increased muscle mass upon testosterone treatment while there was a loss in grip strength. This indicates that muscle mass is not controlled by AR in the myofiber or satellite cell but muscle strength is directly affected by androgen signaling. An RNA seq of the mARKO suggests that glucose/amino acid metabolism and muscle sarcomeric gene regulation, such as *Mylk4*, is regulated by androgen signaling (39).

Aside from satellite cells, a population of uncommitted progenitor cells of mesenchymal (40) and adipocytes origin (41) acts as reservoir of satellite cells during muscle regeneration or hypertrophy. Ar deletion targeted to mesenchymal stem cells resulted in upreglation of key adipogenic transcription factors such as Peroxisome proliferator-activated receptor gamma (*PPARγ*) and CCAAT/enhancer binding protein alpha (*C/EBPα*) (42). Additionally, testosterone treatment of CH3 10T1/2 cells result in the upregulation of myogenic differentiation markers such as *MyoD* and myosin heavy chain II (MyHC) and downregulation of markers for adipogenic differentiation such as *PPARγ* and *C/EBPα* (43). These studies suggest that androgen can shift the commitment of cells from adipocytes or vascular fate to myogenic cells (44). Other cells found in the interstitial compartment such as connective tissues may play a role in regulation of muscle development. A ligament known as gubernaculum which connects the gonad to the future inguinal canal is known to work with the cremaster muscle in the proper descent of the testis. Gubernaculum-specific *Ar* deletion resulted in increased *Pax7* expression and decreased expression of skeletal muscle actin suggesting defective development of the cremaster muscle. However, cremaster muscle-specific *Ar* deletion did not show any observable phenotypes. The authors suggested that the deficiency in AR results in the loss of Ar-induced paracrine signals from the gubernacular cells to the myoblasts (45).

Interestingly, another non-myocytic cell is found to be necessary for the sexually dimorphic development of muscles in the perineum, the BC/LA muscle. The BC is a striated muscle complex inserted into the urethral bulb or bulb of the penis (46, 47) which possesses a posterior extension which is the LA. The functions of these muscles in mouse have not yet been completely studied but equivalent structures in human are suggested to be involved for sustaining erection (BC) and defecation (LA) (48–51). BC/LA complex is known to be sensitive to androgen signaling (52) which is evident in the table, BC/LA is the muscle complex that is mostly affected by muscle-specific AR deletion. A study on the embryonic development of the BC revealed that the *Ar* expressed in the mesenchyme surrounding the BC muscle cells are more important for masculinized BC and not the *Ar* in the myoblast (53). The study mentioned a possible paracrine regulation from non-myocytic cells to myoblast that controls proliferation of myoblast leading to a bigger BC in male. Such well-developed BC in male was feminized after mesenchyme-specific *Ar* deletion.

The second point in studying the sexually dimorphic organ is to identify the inherent difference between male and female muscle development. There are a few studies that compare the development of muscles between male and female or even to compare the difference in muscle markers or gene players in the normal development of sexually dimorphic muscles. The formation of BC and LA are more prominent in male than in female and ArKO mouse exhibits defective formation in such muscles (46, 51). A study discovered that there are more satellite cells in male LA as compared to female and prenatal exposure of female to exogenous testosterone analogue resulted in the increase of satellite cells and increased LA muscle mass. Additionally, a study observed that the formation of BC in male and female was similar until E.16.5 when the myoblast number in males become more prominent than in the female. ArKO in the mesenchyme of the BC resulted into feminized BC muscles (53). In a later stage (E17), the BC/LA of the female undergoes higher apoptosis rate which leads to an underdeveloped perineum muscle in female (51). These studies show that examining development of BC/LA muscles at embryonic stage,while comparing male and female, can provide a clear picture on the role of androgen in the differential muscle mass of male and female BC/LA.

The male and female difference in the development of other muscle groups has not been studied at embryonic stages or whether a sensitive time window of the sexually dimorphism of muscle exists. Many publications show the control of androgen on varying processes of myogenesis (CITE here) but not on the inherent difference between male and female muscle properties. There are some reports of differences in myogenic properties, and its dependency on androgen signaling between males and females that were observed in adult mice. It was identified that global-KO of *Ar* affected only the muscle mass of male mice and not that of the female. A reduction in the muscle mass of the tibialis anterior (TA) extensor digitorum longus (EDL), gastronomicus (GAST), and soleus (SOL) was evident in male ArKO mice and the same muscles were unaffected in female ARKO (54). Furthermore, muscle contractile strength was also reduced in the EDL of male ARKO, becoming more similar to that of the female. There are studies suggesting that the type of fibers between male and females are inherently different from each other. Muscle fiber types is distinguished by the (MyHC) expressed in the fiber and is correlated with the morphology and abundance of glycolytic enzymes. This translates into contractile velocity of the muscles with type IIB having a higher relative velocity than IIA (55) (Resnicow 2010). In mouse masseter, or muscles of mastication, the female has more Type IIA fibers (expressing MyHC-IIA) while male exhibit more type IIB fibers. The expression of MyHC-IIA is higher in males in the SOL and TA but in the plantaris, IIA is higher in females than in males (56). Another study reported that Mylk4 is differentially expressed in fast-type muscles of male and female mice. *Mylk4* is a gene implicated with increased muscle strength and it was found to be more expressed in male fat-type muscles compared to female. Additionally, *Mylk4* expression can be stimulated by androgen treatment (39).

The third perspective on GT development, confirm the androgen-responsive nature of the genes that were found to be inherently different between male and females, is more difficult to be applied in this review. There are numerous studies measuring the androgen responsiveness of varying key players on myogenesis and muscle physiology,which can be found elsewhere (57–59). These reports are extremely helpful in understanding the possible use of androgens on muscle dystrophy and muscle regeneration. These may be used as initial candidates in understanding the inherent difference in male and female muscles. This will require examining the role of these genes or processes in the natural setting of male and female muscle development and growth.

## Conclusion

In terms of musculature, are males and females created equal? The investigation on the sexual dimorphism of the muscles could utilize the perspectives used in discovering the formation of a gender-specific ExG. It would be helpful to identify what are the unique properties of male and female muscles and identify whether these properties are responsive to androgen. It is also important to recognize the different tissues or cells involved in the development and/or physiology of muscles. Each cell type might contribute to the muscle growth through autocrine and paracrine fashion. However, the muscle, as an organ, has its own complexity different from the ExG. Muscles have different muscle fiber types, with each muscle complex comprised of different ratio of such fiber types. These contributes to the differential androgen responsiveness/dependency of numerous muscle groups. Unlike the ExG, muscles can regenerate and such process, its fruition and implementation, adds another vantage point in understanding androgen`s influence. Muscles has many other properties that goes beyond structure such as muscle strength, contractile velocity, and endurance which cannot be easily distinguished as dimorphic or not. This makes the study of muscle development dynamic and,dare I say, exciting.

## Author contributions

The author confirms being the sole contributor of this work and has approved it for publication.

## Conflict of interest

The author declares that the research was conducted in the absence of any commercial or financial relationships that could be construed as a potential conflict of interest.

## Publisher’s note

All claims expressed in this article are solely those of the authors and do not necessarily represent those of their affiliated organizations, or those of the publisher, the editors and the reviewers. Any product that may be evaluated in this article, or claim that may be made by its manufacturer, is not guaranteed or endorsed by the publisher.

## Supplementary material

Supplementary Table 1 A summary of studies detailing the effect of androgen on muscle as mediated by different cell population found in the muscles.
