# Supplementary material for: Sexual dimorphism through androgen signaling; from external genitalia to muscles
Source: Front Endocrinol (Lausanne). 2022 Jul 27;13:940229. doi: 10.3389/fendo.2022.940229 (PMC9379613; doi:10.3389/fendo.2022.940229)
Supplement: Supplementary Table 1 — A summary of studies detailing the effect of androgen on muscle as mediated by different cell population found in the muscles. [file Table_1.docx]

Supplementary Table 1. A summary of studies detailing the effect of androgen on muscle as mediated by different cell population found in the muscles.

| Androgen Receptor Knock-out | Affected cell/ muscles examined | Effects | References | |
| --- | --- | --- | --- | --- |
| MCKCre^+/−^;AR^fl^/Y | Myocyte or myofiber/quadriceps, extensor  digitorum longus (EDL), gastronomicus (GAST), soleus (SOL) , LA muscles | Muscle mass reduced in LA and EDL  SOL shift to more type I fibers | Ophoff et al., 2009 |  |
| HSA-AR/Tfm rats (gof of AR in musclesonly) | Muscles/LA | Rescue of AR on Tfm mice did not change the LA volume | Niel et al., 2009 | |
| AR^fl^/Y;MyoD-iCre+^/-^ | Satellite cells and descendant myoblast (MyoD-expressing cells)/SOL, EDL, LA | Decreased grip strength; SOL shift from type I fibers.; LA muscle mass decreased | Dubois et al., 2014 | |
| MCK mAR^ΔZF2^ | Post-proliferative myofibers/  EDL,TA,SOL,GAST, LA | Muscle mass of EDL,TA,SOL, LA reduced | Rana et al.,2016 | |
| mAR^ΔZF2^ | Myoblasts/ EDL,TA,SOL,GAST, LA | ONLY LA muscle mass reduced | Rana et al.,2016 | |
| Pax7^CreERT2^+;AR^flox^ | Satellite cells/ tibialis anterior (TA) muscles | Muscle mass still increased in the presence of testosterone treatment | Sakakibara et al., 2021 | |
| Ar^flox/flox^;HSA-Cre | Muscle fiber/ TA | Muscle mass still increased in the presence of testosterone treatment but grip strength was reduced | Sakakibara et al., 2021 | |
| Ar^flox/flox^;Rarb-cre | Gubernaculum cells/Cremaster muscle | Abnormal cremaster muscle development | Kaftanovskaya et al., 2012 | |
| Sall1-Cre^ER/+^ AR^flx/Y^ | Mesenchyme of BC/BC muscles | Female-like BC muscle development | Ipulan et la., 2014 | |
